# The Synthesis and Characterization of Selected Optically Active Sulfoxides Bearing Perfluorocumyl Moiety, Their Spontaneous Reversible Transformations into Higher-Valent Organosulfur Species–Bicyclic Hydroxysulfuranes, and Their Irreversible Transformation into Sultine

**DOI:** 10.3390/molecules31060969

**Published:** 2026-03-13

**Authors:** Adrian Zajac, Ewelina Wielgus, Józef Drabowicz

**Affiliations:** 1 Division of Organic Chemistry, Center of Molecular and Macromolecular Studies, Polish Academy of Sciences, Sienkiewicza 112, 90-363 Łódź, Poland; adrian.zajac@ppnt.poznan.pl; 2 Materials Synthesis Group, Poznan Science and Technology Park, Rubiez 46, 81-812 Poznan, Poland; 3 Division of Structural Chemistry, Center of Molecular and Macromolecular Studies, Polish Academy of Sciences, Sienkiewicza 112, 90-363 Łódź, Poland; ewelina.wielgus@cbmm.lodz.pl; 4 Department of Chemistry, Environmental Protection and Biotechnology, Jan Dlugosz University in Czestochowa, Armii Krajowej 13/15, 42-201 Czestochowa, Poland

**Keywords:** sulfoxide, sulfurane, hypervalent bond, sultine, optical activity, 1,1,1,3,3,3-hexafluoro-2-phenylpropan-2-ol, perfluorocumyl alcohol, ortholithiation, Andersen methodology, NMR spectroscopy, circular dichroism

## Abstract

The preparation of the novel optically active sulfoxides (-)-(*S*)-1,1,1,3,3,3-hexafluoro-2-[*o*-(*p*-tolylsulfinyl)phenyl]propan-2-ol **1**, (-)-(*S*)-1,1,1,3,3,3-hexafluoro-2-[*o*-(methylsulfinyl)phenyl]propan-2-ol **2** and (-)-(*S*)-1,1,1,3,3,3-hexafluoro-2-[*o*-(*t*-butyl-sulfinyl)phenyl]propan-2-ol **3** according to the Andersen methodology and their spectroscopic characterization is presented. The NMR and CD spectroscopic evidence of the existence of the equilibrium between sulfoxide and hypervalent sulfurane forms of these compounds in solution and attempts at the isolation of corresponding sulfuranes are shown. For compound **3**, the unprecedented subsequent irreversible transformation in solution into corresponding cyclic sulfinate ester–sultine **17** was established on the basis of NMR spectroscopy measurements. The mechanism of this transformation was investigated by means of GC-MS analysis and confirmed on the basis of synthesized long alkyl chain analog **23** transformation in solution. Moreover, the oxidation properties of obtained sulfoxides **2** and **3** for the selected compounds are described.

## 1. Introduction

Sulfuranes, compounds with a central sulfur atom of higher than formal valency and a 3c-4e hypervalent bond, have been known for a relatively long time [1,2]. Their chirality, especially in the case of spirosulfuranes, is also well documented [2,3,4,5,6]. Although there are a few publications presenting stable chiral bicyclic sulfuranes bearing a strongly electronegative halogen atom as an apical substituent [3,7,8,9,10,11], examples of that kind of stable sulfurane with a hydroxyl group in an apical position have not been shown till now. Selected sulfoxides bearing perfluorocumyl moiety seem to be appropriate precursors for obtaining the hydroxysulfuranes mentioned above, because of the presence of two strongly electron-withdrawing perfluoromethyl groups and the phenyl ring, together with the possibility of forming a five-membered ring containing “a half” of a hypervalent bond, the properties of which can stabilize the desired hypervalent structure. Furthermore, those sulfinyl compounds could be useful as bidental chiral ligands and chiral auxiliaries.

## 2. Results and Discussion

Herein, we present results of studies on the synthesis and behavior in solution of novel optically active sulfoxides bearing a perfluorocumyl moiety: (-)-(*S*)-1,1,1,3,3,3-hexafluoro-2-[*o*-(*p*-tolylsulfinyl)phenyl]propan-2-ol **1**, (-)-(*S*)-1,1,1,3,3,3-hexafluoro-2-[*o*-(methylsulfinyl)phenyl]propan-2-ol **2** and (-)-(*S*)-1,1,1,3,3,3-hexafluoro-2-[*o*-(*t*-butyl-sulfinyl)phenyl]propan-2-ol **3**.

**Scheme 1:**
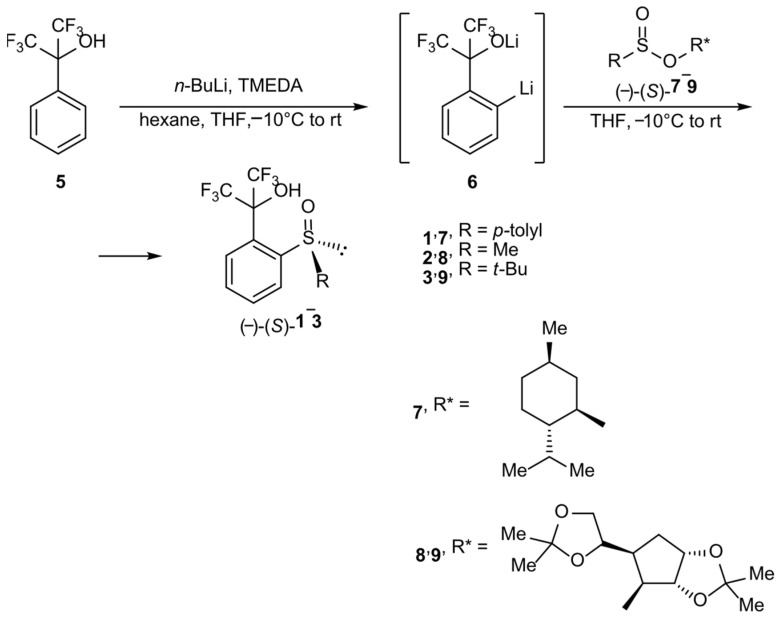
Synthesis of sulfoxides **1**–**3**.

They were obtained according to the Andersen methodology [12] using an ortholithiation reaction of perfluorocumyl alcohol **5** and by treating formed in situ dilithio-derivative **6** with corresponding optically pure sulfinate esters **7**, **8** or **9** (Scheme 1). The reactions occur with the inversion of the absolute configuration on a sulfur atom. In the case of sulfoxide **1**, the substrate was commercially available *O*-(-)-(1*R*,2*S*,5*R*)-menthyl (-)-(*S*)-*p*-toluenesulfinate **7**, and for compounds **2** and **3**, *O*-diacetone-*D*-glucosyl (-)-(*S*)-methanesulfinate **8** and *O*-diacetone-*D*-glucosyl (-)-(*S*)-*t*-butanesulfinate **9**, respectively. Compounds **8** and **9** were obtained using a method presented elsewhere [13].

The choice of *O*-diacetone-*D*-glucosyl sulfinate esters for the synthesis of compounds **2** and **3** instead of corresponding *O*-(-)-(1*R*,2*S*,5*R*)-menthyl derivatives was dictated by better yields and stereoselectivity. Nevertheless, the yields of the presented processes were rather low; sulfoxide **1** showed the highest yield value, of 40%, but for **2,** this was only 16%, probably because of the side reaction of deprotonation of the methyl group bonded to the sulfur atom by dilithio-derivative **6**. The lowest yield was for **3**, at 7%, which was most likely due to the bulkiness of the *t*-butyl group. The unsatisfactory yields were compensated by very high stereoselectivity. Product **1** was obtained with 95% ee, and in the case of **2** and **3,** the process was stereospecific.

**Figure 1:**
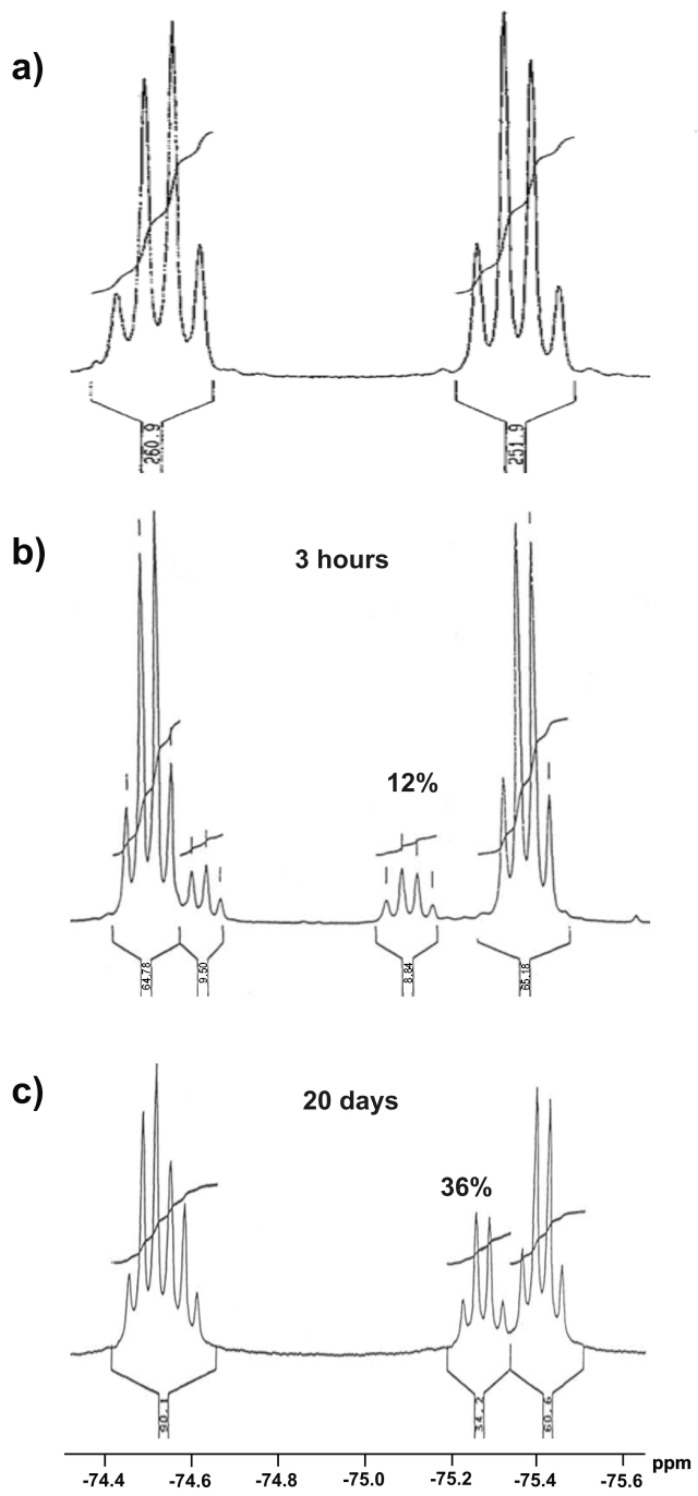
Series of ^19^F NMR spectra of sulfoxide **1** in CDCl_3_: (**a**) after isolation, (**b**) after 3 h in solution, (**c**) after 20 days in solution.

**Scheme 2:**
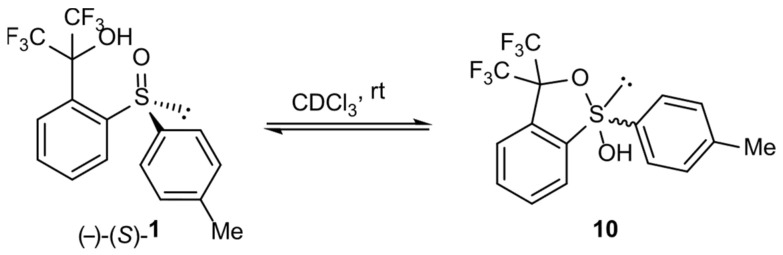
Reversible transformation of sulfinyl derivative **1** into hydroxysulfurane structure **10** in solution.

In order to prove the possibility of transformation of sulfoxide **1** form into corresponding hydroxysulfurane **10**, compound **1** was crystallized from DCM to give monocrystal. However, its X-ray diffraction analysis allowed us to unambiguously establish that the crystallized compound had a sulfoxide structure with the *S* absolute configuration on the sulfur atom. On the other hand, the series of ^19^F NMR spectra of compound **1** solution in CDCl_3_ was recorded. They showed two quartets characteristic of nonequivalent perfluoromethyl groups as a result of the near presence of the chiral sulfinyl group (Figure 1a). The prolongation of the experiment to 3 h resulted in the appearance of an additional pair of quartets of lower intensity (Figure 1b), which suggested the spontaneous formation of another chiral structure, hydroxysulfurane **10**. After 20 days, the intensity of “new” signals increased from 12% to 36% at the expense of the “old” pair (Figure 1c), which, in turn, meant the continuous conversion of sulfoxide **1** to hypervalent structure **10**. Attempts to isolate compound **10** from that experiment failed and gave sulfoxide **1** as the only product. These observations proved the existence of the spontaneous and reversible transformation of sulfinyl derivative **1** into hydroxysulfurane structure **10** (Scheme 2). The change in chemical shifts of the quartet signals in Figure 1b,c can be attributed to a change in the chemical composition of the solution and, thus, the change in the intensity of intermolecular interactions between **1** and **10**, especially if we take into consideration the presence of hydroxyl groups (hydrogen bond donors) and fluorine atoms (hydrogen bond acceptors), which can interact with each other relatively strongly.

**Figure 2:**
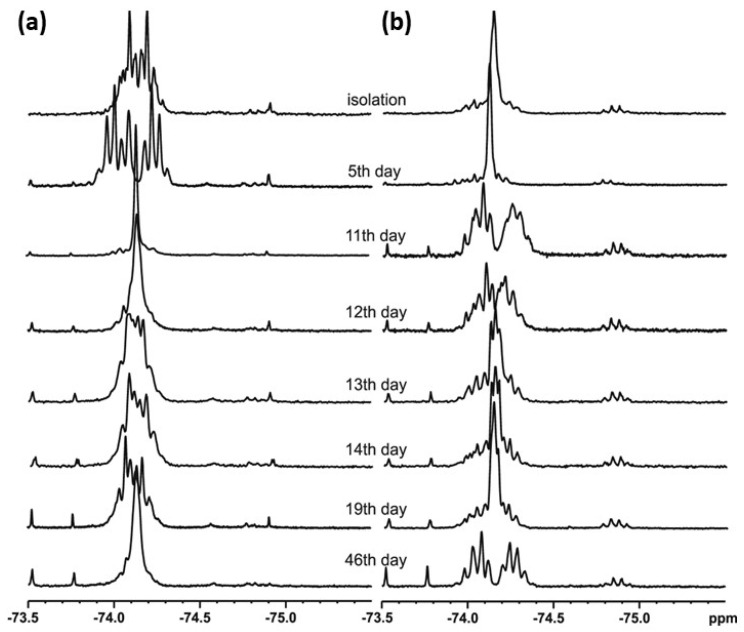
The ^19^F NMR spectra in CDCl_3_ of: (**a**) fraction 1, (**b**) fraction 2.

**Figure 3:**
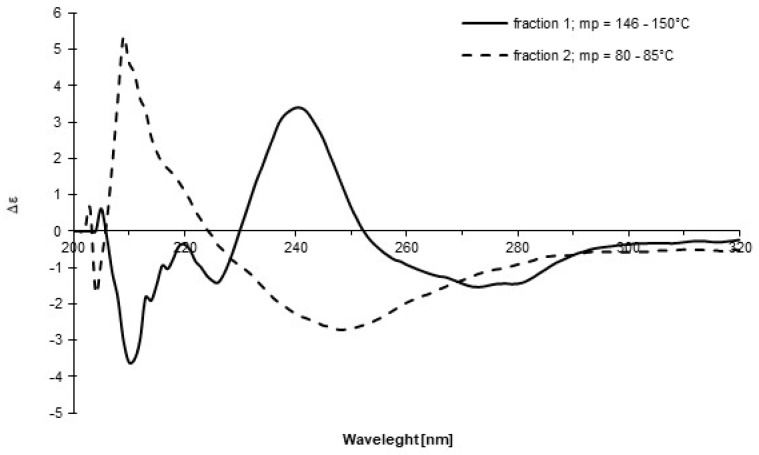
The CD spectra of two fractions obtained during purification process of **1** recorded in DCM.

We repeated the above synthesis, and, during purification using column chromatography, two groups of fractions were isolated based on TLC analysis. The ^19^F NMR spectra in CDCl_3_ for each group were recorded at time intervals (Figure 2). The experiment clearly showed intense dynamics in both systems, manifested by the cyclic coalescence and differentiation of the quartet signals mentioned above. This result is further evidence of the existence of structures **1** and **10** in each fraction and their dynamic reversible transformation into one another. The formation of two unidentified products (indicated by the appearance of two singlet signals) was also observed. Moreover, for both groups of fractions, CD spectra in DCM were recorded (Figure 3) and showed opposite Cotton effects with shifted maxima relative to each other, and after isolation, their melting points were significantly different (146–150 °C and 80–85 °C), which confirmed the presence of two different chiral structures: sulfoxide **1** and sulfurane **10**. Unfortunately, all attempts to crystallize either form did not furnish a signle crystal of sufficient quality for X-ray diffraction analysis. The differences between Figure 1 and Figure 2 can be explained in terms of the significantly different sample histories. In Figure 1, the sample is pure sulfoxide **1** after its preparation, while in Figure 2, two groups of fractions with not precisely established composition are analyzed, which can affect parameters such as chemical shifts. We assume that the driving forces of the transformation of compound **1** into **10** are as follows: (a) intramolecular coordination of the hydroxyl to sulfur; (b) formation of a stabilizing 3c–4e hypervalent bond, which likely requires stabilization by the solvent, as we were unable to isolate this species; (c) the strong electron-withdrawing effect of the perfluorocumyl moiety stabilizing the hypervalent bond; (d) formation of a five-membered ring, which reduces the entropic penalty. On the other hand, the reverse transformation, from **10** to **1**, is most probably driven by reaching a certain concentration limit of **10** in solution, followed by destabilizing intermolecular interactions.

**Figure 4:**
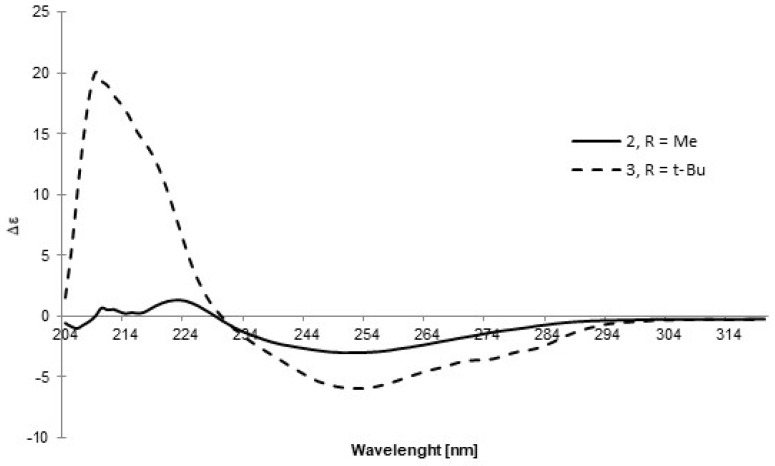
The CD spectra of sulfoxides **2** and **3** recorded in DCM.

A similar approach was applied to the other two sulfoxides, **2** and **3**. Due to difficulties in crystallizing these compounds, the absolute configuration was determined using an indirect comparative method. CD spectra of these compounds were recorded in DCM (Figure 4) and compared with the spectra of analogous sulfoxides, (*R*)-*p*-tolyl-methylsulfoxide and (*R*)-*p*-tolyl-*t*-butylsulfoxide, respectively, as reported by Mislow et al. [14]. The opposite signs of the Cotton effect at similar wavelengths together with the assumption that the reaction gives a product of inverted configuration with respect to precursors **8** and **9** allowed for the conclusion that sulfoxides **2** and **3** possess the *S_S_* absolute configuration.

**Scheme 3:**
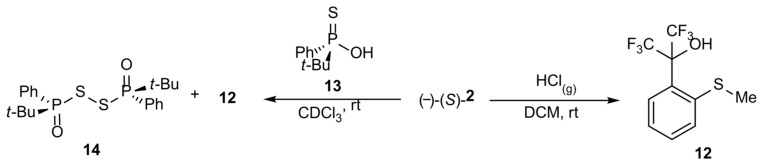
Oxidizing effect of sulfoxide **2**.

As previously, the enantiomeric excess was determined via the HPLC technique using a column with a chiral stationary phase and racemic mixtures of compounds **2** and **3** as a reference. It is noteworthy that racemization attempts with gaseous HCl in DCM at rt for (-)-(S)-*t*-butylsulfoxide **3** resulted in the corresponding racemate but only in a 10% yield, whereas the main product was sulfide **11**, the result of the reduction of starting sulfoxide **3**, obtained in a 90% yield (Equation (1)). In the case of compound **2**, the corresponding sulfide **12** was the only product (Scheme 3). These conclusions were confirmed by ^19^F NMR spectra. These are examples of sulfoxide reduction by gaseous HCl. Moreover, attempts to determine the ee of methylsulfoxide **2** by the use of the chiral solvating agent—*t*-butylphenylthiophosphinic acid **13**—in CDCl_3_ resulted in the oxidation of compound **13** to disulfide **14,** with the simultaneous reduction of **2** to **12** (Scheme 3) according to ^31^P and ^19^F NMR spectra, which indicates that a sulfoxide bearing the perfluorocumyl moiety is a stronger oxidant than analogous aryl methyl sulfoxides.
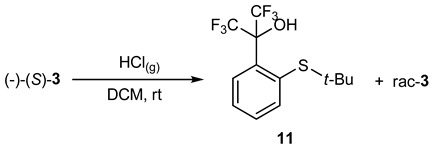
(1)

**Figure 5:**
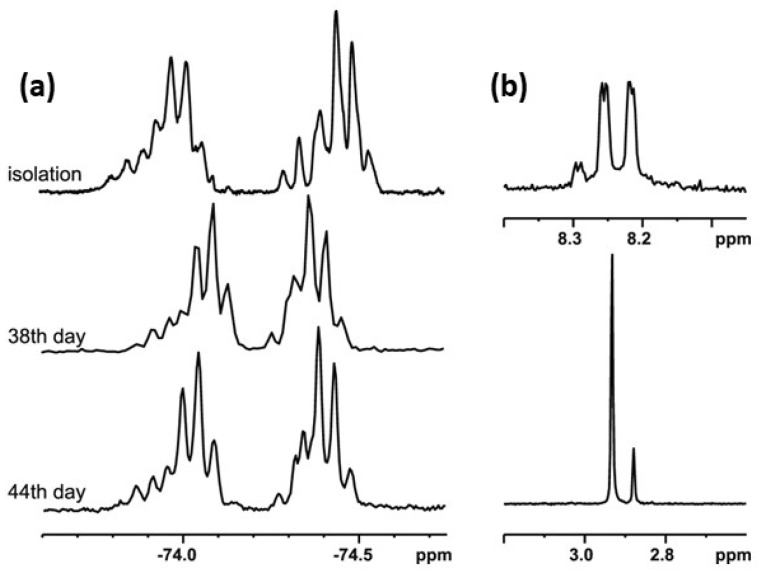
The NMR spectra of compound **2**: (**a**) ^19^F NMR, (**b**) ^1^H NMR (selected regions).

**Scheme 4:**
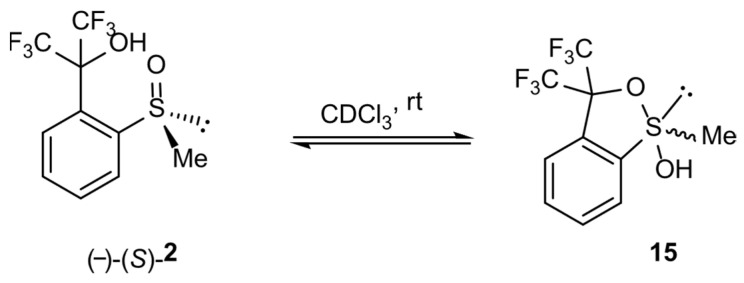
Equilibrium between sulfinyl **2** and sulfurane **15** structures in solution.

In order to check the possibility of the transformation of alkyl derivatives **2** and **3** into the corresponding hypervalent structures **15** and **16**, respectively, similar long-lasting NMR experiments were conducted. The series of ^19^F NMR spectra (Figure 5a) confirmed the existence of two different chiral structures in a CDCl_3_ solution. They showed two multiplet signals, which are combinations of two quartet resonance signals each, as in the case of compound **1**. Moreover, the ^1^H NMR spectrum (Figure 5b) recorded for the same solution contained doubled resonance signals related to the protons of the methyl group bonded to the sulfur atom (2.88 and 2.93 ppm, Figure 5b, lower image) and to the aromatic proton at the meta position of the benzene ring (8.2–8.3 ppm region, Figure 5b, upper image). The spectrum remained unchanged until the end of the experiment. The analysis of the spectra indicated an equilibrium between the sulfinyl **2** and sulfurane **15** structures in solution (Scheme 4).

**Figure 6:**
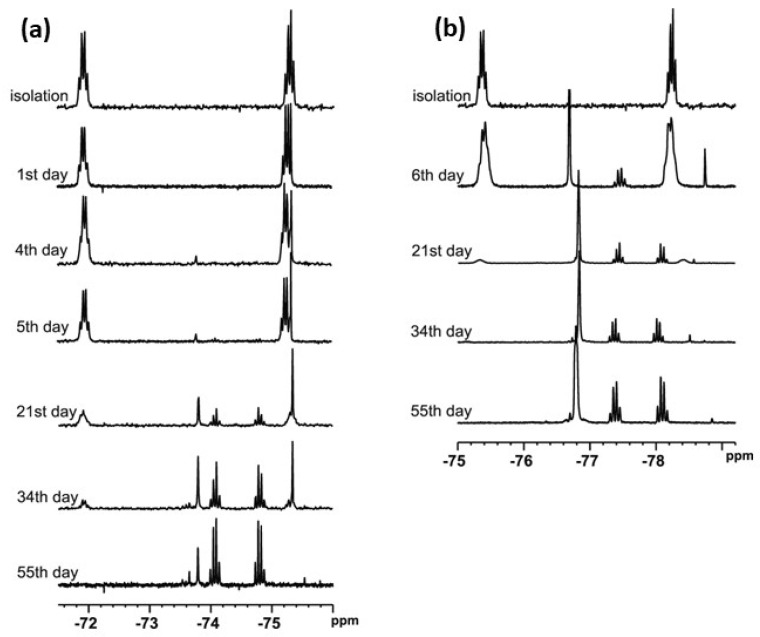
The ^19^F NMR spectra of compound **3** recorded at time intervals in: (**a**) CDC_l3_, (**b**) DCM.

**Figure 7:**
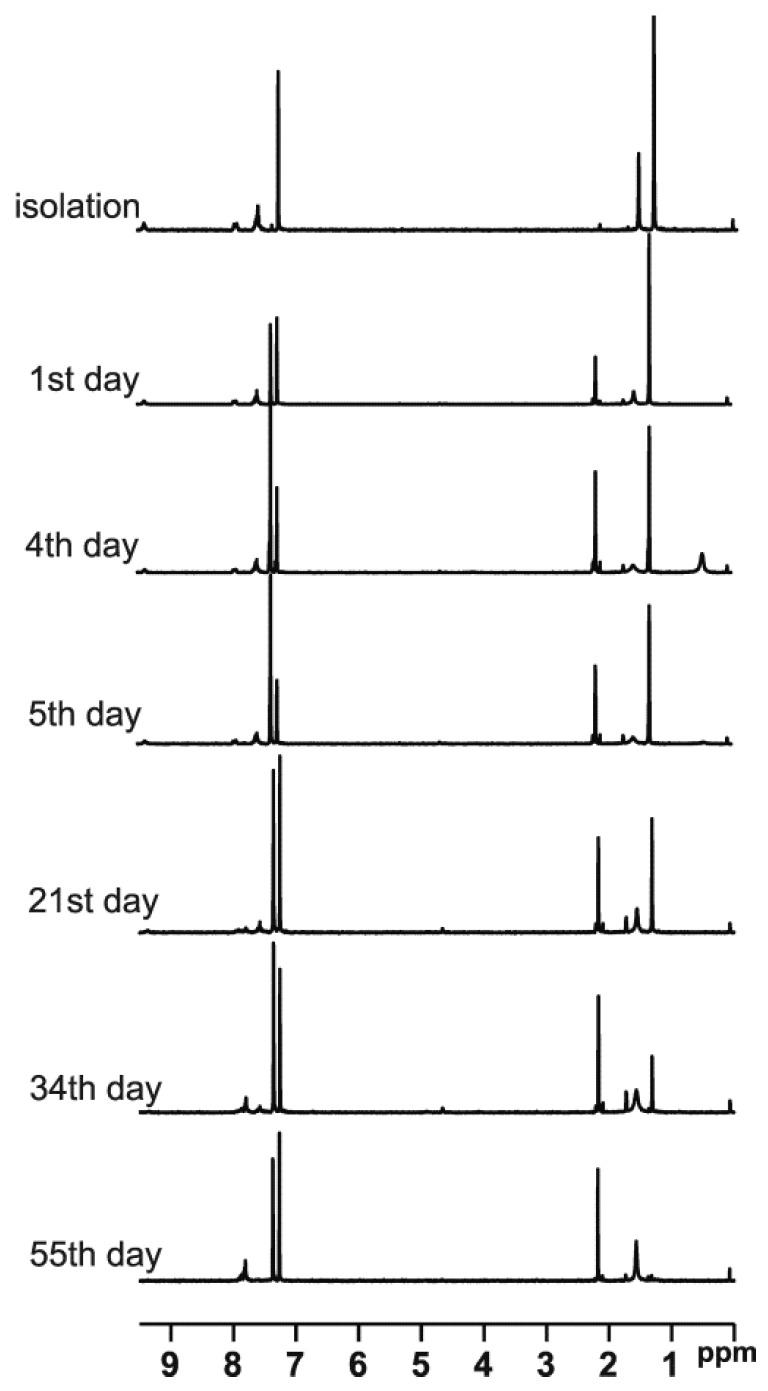
The ^1^H NMR spectra of compound **3** recorded at time intervals in CDC_l3_.

**Scheme 5:**
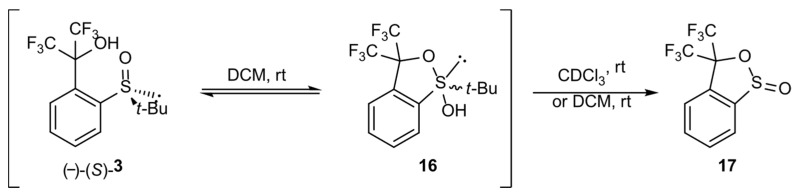
Transformations of sulfoxide **3** in solution.

*t*-Butylsulfoxide **3** turned out to be the most interesting among the presented compounds. Three sets of spectra at time intervals were recorded: ^19^F NMR in CDCl_3_ (Figure 6a) and in DCM (Figure 6b), and ^1^H NMR in CDCl_3_ (Figure 7). The existence of both sulfoxide **3** and hydroxysulfurane **16** (Scheme 5) in solutions was confirmed by the broadening of the quartet resonance signals in the ^19^F NMR spectrum in DCM after 6 days (Figure 6b), and the presence of a doubled singlet signal referred to the protons of the *t*-butyl group in the ^1^H NMR spectrum after isolation (1.32 and 1.56 ppm, Figure 7). Interestingly, compound **3** underwent another irreversible transformation into a structure with a stereogenic sulfur atom. It was evidenced by the appearance of a new pair of quartet signals at −74.80 and −74.04 ppm in the ^19^F NMR spectra (Figure 6), both in CDCl_3_ and DCM, which was accompanied by the disappearance of the signal of the *t*-butyl group protons. These observations, supported by the mass spectrometry analysis [MS EI, *m*/*z* = 290.0 (100%)] and comparisons with the data acquired for the product obtained in an independent experiment, allowed for the identification of the product formed from sulfoxide **3** as the cyclic sulfinate ester **17** (Scheme 5). The formation of **17** was preceded by the production of two unidentified achiral products, which was indicated by the appearance of two singlet signals (−75.33 and −73.79 ppm) in the ^19^F NMR spectra (Figure 6a). Due to the decrease in the intensity of one of them (−75.33 ppm), it was probably assigned to the precursor of compound **17**.

**Scheme 6:**
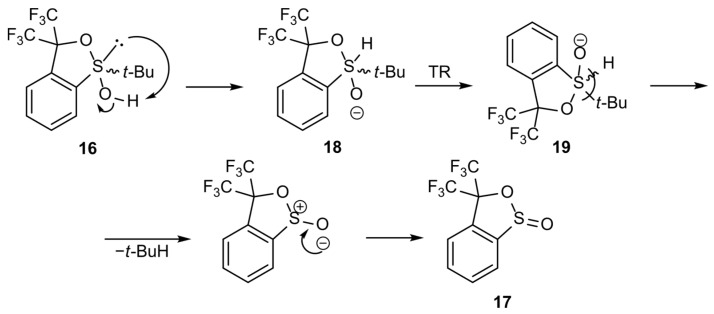
Hypothetical mechanism of sulfurane **16** transformation into sultine **17**.

Two hypothetical alternative mechanisms were proposed. According to the first one, existing in equilibrium with the sulfoxide structure **3**, sulfurane **16** underwent rearrangement to another sulfurane **18,** followed by turnstile rotation (TR) [1,2] of the latter to give structure **19**. Sulfurane **19** underwent a ligand coupling reaction to furnish sultine **17** and 2-methylpropane as a side product (Scheme 6). The mechanism would constitute an explanation for the formation of ester **17** in DCM solution.

**Scheme 7:**
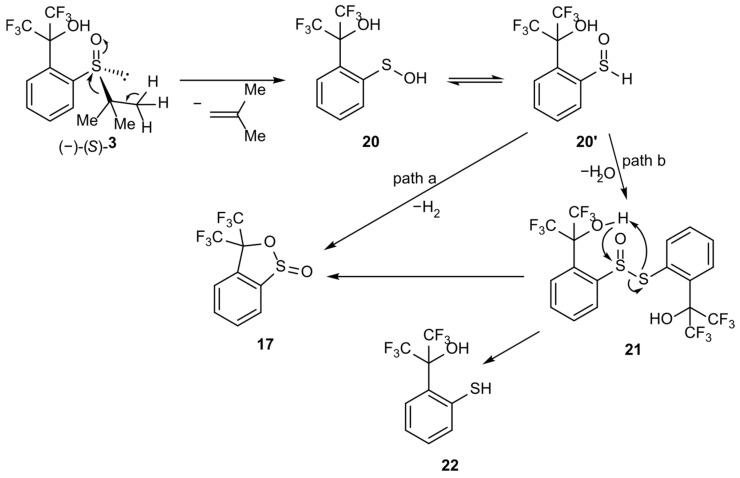
Hypothetical mechanism of sulfoxide **3** transformation into sultine **17**.

The second mechanism could be more appropriate for the CDCl_3_ solution of **3**. It assumes the initial elimination of 1,1-dimethylethylene from sulfoxide **3** and the formation of sulfenic acid **20**. Acid **20** furnishes sultine **17** either via the direct elimination of a hydrogen molecule from the tetravalent tautomer **20′** and cyclization (Scheme 7, path a) or its dimerization to thiosulfinate **21** (Scheme 7, path b) and subsequent cyclization as a result of the nucleophilic attack of the oxygen atom in the hydroxyl group on the sulfinyl sulfur atom (Scheme 7). Path b requires the formation of thiol **22,** whose existence could explain the appearance of one of the singlet resonance signals in the ^19^F NMR spectrum.

**Scheme 8:**
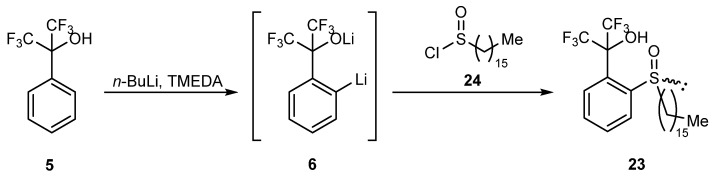
Synthesis of sulfoxide **23**.

In order to confirm one of the proposed mechanisms, recordable products of the described transformation were required. That goal was achieved through the synthesis of racemic analog **23** bearing a long alkyl substituent with the use of hexadecanesulfinyl chloride **24** (Scheme 8). The reaction was carried out by ortho-lithiation of alcohol **5**, followed by treatment of the resulting mixture with an equimolar amount of chloride **24** in THF at room temperature for 22 h. The obtained crude product was then subjected to column chromatography followed by preparative thin-layer chromatography to give pure product **23** in a low yield (10%).

**Figure 8:**
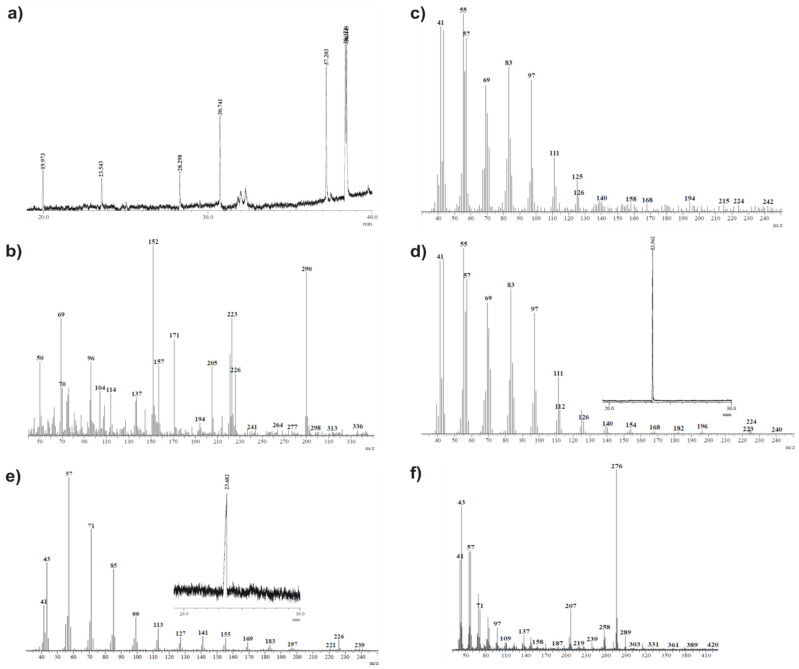
The GC-MS analysis results obtained for compound **23** kept in CDCl_3_ solution for 72 days and corresponding reference materials: (**a**) chromatogram of **23** solution after 72 days, (**b**) MS profile of 19.97 min. GC peak, (**c**) MS profile of 23.54 min GC peak, (**d**) MS profile of 23.56 min. GC peak recorded for commercial 1-hexadecene, serving as reference, (**e**) MS profile of 23.57 min. GC peak recorded for commercial hexadecane, serving as reference, (**f**) MS profile of 38.44 min. GC peak.

The sample of the obtained compound was kept in a CDCl_3_ solution at room temperature for 72 days (until the complete disappearance of the initial quartet signals in the ^19^F NMR spectrum), and the resulting mixture was then subjected to GC-MS analysis. According to both proposed mechanisms, the main product of the discussed transformation would be sultine **17**, but in the first case (Scheme 6), it would be accompanied by the corresponding saturated hydrocarbon and, in the second case (Scheme 7), by the corresponding unsaturated hydrocarbon and probably thiol **22**. In view of the above, based on initial GC-MS analyses of commercially available references, 1-hexadecene and hexadecane were recorded (Figure 8c,e, respectively). As expected, the retention times of these two compounds were similar (23.54 and 23.57 min., correspondingly), but the results of MS analysis differed significantly, excluding the possibility of a mistake. Next, GC analysis of the sample mixture was conducted to give the chromatogram shown in Figure 8a. The obtained peak MS profiles are shown in Figure 8b,c,f. Figure 8b presents the MS profile of the peak with a retention time equal to 19.97 min and clearly indicates the presence of sultine **17** in the mixture (*m*/*z* 290). In Figure 8c, the MS profile of the peak at 23.54 min. is shown. Both the retention time with *m*/*z* values and MS peak intensities are almost identical to those recorded for the 1-hexadecene reference, which indicates the presence of this compound in the analyzed mixture. Moreover, MS analysis of the peak with a retention time of 38.44 min. revealed the presence of thiol **22** (*m*/*z* 276).

On the basis of the above results, it can be claimed that the presented transformation occurs according to the mechanism shown in Scheme 7, path b, as follows:

1. Elimination of a substituent at the sulfur atom with formation of the corresponding alk-1-ene and sulfenic acid **20**. The presence of a hydrogen atom at the *β*-carbon atom and its aliphatic nature is necessary. In the absence of these features, the described process does not occur, as can be observed for compounds **1** (methyl group at the sulfur atom—no *β*-hydrogen atom) and **2** (*p*-tolyl group at the sulfur atom—aromatic *β*-carbon atom).
2. Reversible rearrangement of sulfenic acid **20** into sulfoxide **20′**.
3. Spontaneous condensation of molecules **20** and **20′** into the unstable thiosulfinate **21** with the release of a water molecule.
4. Sultine ring closure in structure **21** leading to the formation of sultine **17** with simultaneous elimination of thiol **22**.

The above-described process seems to be general for every *S*-substituted *o*-sulfinyl derivative of 1,1,1,3,3,3-hexafluoro-2-phenylpropan-2-ol after the fulfilment of the structural restrictions mentioned above. This aspect will be further investigated.

The above results also explain the changes in the NMR spectra of sulfoxide **3**. In Figure 6a, the broadened singlet signal at ~−75.5 ppm can be attributed to unstable thiosulfinate **21** due to the fact that it disappears at the end of the process, although this assignment is not certain. In turn, the sharp singlet signal at ~−73.9 ppm, appearing on day 4–5 of the process and increasing its intensity from that point, can be attributed to the emerging thiol **22** (the chemical shift is in acceptable agreement with the literature data [15]). Obviously, the initial disappearance of the first pair of quartet signals in favor of the second pair of quartets is related to initial sulfoxide decomposition and the formation of the final sultine **17**. An analogous situation can be observed in Figure 6b, though with different chemical shifts due to the lack of lock during recording of the spectra. In the ^1^H NMR spectra evolution, some newly formed signals can also be observed. New signals in the aliphatic region can be attributed to the formation of the alkene—1,1-dimethylethylene—partially soluble in CDCl_3_, while two new groups of signals in the aromatic region can be attributed to sultine **17** and thiol **22**.

## 3. Materials and Methods

For column chromatography, silica gel 60 (70–230 mesh) (Merck, Darmstadt, Germany) was used. The NMR spectra were obtained using 200 MHz and 600 MHz spectrometers (Bruker, Billerica, MA, USA). The IR spectra were recorded using FTIR spectrometer (Bruker, Billerica, MA, USA) via the KBr tablet method. The mass spectra and HRMS were measured using double-focusing (BE geometry) mass spectrometer (Thermo Fisher Scientific, Waltham, MA, USA) utilizing chemical ionization (CI) method with isobutane as an ionizing agent or electron ionization (EI) technique. The melting point was measured using capillary apparatus (Electrothermal, Rochford, UK). The specific optical rotation values were measured with the use of a polarimeter (Anton Paar, Graz, Austria) at wavelength of 589 nm and room temperature. The enantiomeric excesses were determined using HPLC technique (Varian, Palo Alto, CA, USA) on the analytical column with a chiral stationary phase and corresponding racemic mixtures as a reference. The CD spectra were recorded with circular dichroism spectrometer (JASCO, Tokyo, Japan). The GC-MS analysis was conducted at injection temperature of 200 °C in split mode (split ratio 1:40), and the analyte separation was achieved at column gas flow equal to 1.30 mL/min and the following oven temperature program: initially, 35 °C for 5 min; then, the temperature was increased to 300 °C within 8 min. and kept at this level for next 10 min. The MS analysis of separated fractions was realized using electrospray ionization (ESI) technique.

(-)-(*S*)-1,1,1,3,3,3-Hexafluoro-2-[*o*-(*p*-tolylsulfinyl)phenyl]propan-2-ol (1); General procedure: [15]

Into a stirred-in-argon-atmosphere solution of *n*-BuLi in hexane (2.5 M, 10 mL, 25.0 mmol), TMEDA was added dropwise (0.57 mL, 3.75 mmol, 15%mol with respect to *n*-BuLi) at rt, and the solution was stirred for further 30 min. After this time, the mixture was cooled to −10 °C and a solution of perfluorocumyl alcohol **5** (2.08 g, 8.50 mmol) in dry THF (5 mL) was added dropwise for 5 min. Next, the mixture was allowed to warm up to rt and stirred for 24 h. After that, the solution was cooled to −10 °C and a solution of (-)-(*S*)-menthyl *p*-toluenesulfinate **7** (2.35 g, 8.00 mmol) in dry THF (10 mL) was added dropwise for 10 min. The obtained solution was allowed to warm up to rt and stirred for 24 h. Next, the reaction mixture was quenched with H_2_SO_4_ aqueous solution (5%; 5 mL) and H_2_O (20 mL), and the obtained two-phase system was extracted with DCM (4 × 30 mL); organic layers were combined, dried over MgSO_4_, and the solvent was evaporated. The obtained crude reddish oil was purified using column chromatography (SiO; Et_2_O/petroleum ether 1:1, *v*/*v*) to furnish pale-yellow solid (1.154 g, 40%).

${\left[\alpha \right]}_{D}^{298}$ = −95.8 (*c* 1.22; DCM); ee = 95% [HPLC; Chiralpak AS; hexane/(iPrOH/EtOH 4:1, *v*/*v*) 9:1, *v*/*v*; flow rate 0.5 mL/min]; mp = 148–150 °C; R*_f_* = 0.6 (Et_2_O).
^1^H NMR (200 MHz, CDCl_3_): *δ* = 8.44 (bs, 1H), 8.16 (d, 1H, ^3^*J*_H-H_ = 6.7 Hz), 7.82–7.37 (m, 5H), 7.19 (d, 2H, ^3^*J*_H-H_ = 1.7 Hz), 2.34 (s, 3H).
^13^C NMR (50 MHz, CDCl_3_): *δ* = 145.1, 142.6, 141.0, 130.8, 129.6, 128.3, 127.7, 127.3, 126.0, 79.9 (h, ^2^*J*_F-13C_ = 119.3 Hz), 21.2.
^19^F NMR (188 MHz, CDCl_3_): *δ* = −74.11 (q, 3F, ^4^*J*_F-F_ = 9.1 Hz), −74.39 (q, 3F, ^4^*J*_F-F_ = 9.1 Hz).
IR (KBr): 3434 (O-H stretch), 1400 (O-H bend), 1153 (S=O sulfoxide/O-S-O sulfurane), 1130 (C-F stretch), 707 (C-S stretch) cm^−1^.
MS (CI): *m*/*z* (%) = 383.0 (100) [M + H]^+^.
HRMS (EI): *m*/*z* calcd for C_16_H_12_SO_2_F_6_ 382.04622; found 382.04547 (Δ 1.96380).
(-)-(*S*)-1,1,1,3,3,3-Hexafluoro-2-[*o*-(methylsulfinyl)phenyl]propan-2-ol (2)

The synthesis was carried out according to the general procedure with use of the following: *n*-BuLi solution in hexane (2.5 M, 8.40 mL, 21.00 mmol), TMEDA (0.47 mL, 3.15 mmol, 15%mol with respect to *n*-BuLi), perfluorocumyl alcohol **5** (1.71 g, 7 mmol) solution in dry THF (1.5 mL), (-)-(*S*)-*O*-diaceton-*D*-glucosyl methanesulfinate **8** (0.99 g, 3.1 mmol) solution in dry THF (5 mL), aqueous solution of H_2_SO_4_ (5%, 5 mL) and H_2_O (20 mL). The reaction mixture after quenching was extracted with Et_2_O (8 × 20 mL); organic layers were combined, washed with H_2_O, and dried over MgSO_4_. The solvent evaporation furnished crude product as a orange oil (2.43 g). After purification with two subsequent rounds of column chromatography, [SiO_2_; Et_2_O (24 × 20 mL), acetone (17 × 20 mL)] followed by [SiO_2_; acetone/petroleum ether 3:1, *v*/*v* (30 × 20 mL), acetone (6 × 20 mL)], a white solid was obtained (153 mg; 16%).

${\left[\alpha \right]}_{D}^{298}$ = −178.1 (*c* 0.53; CDCl_3_); ee > 99% (HPLC; Chiralpak AS; hexane/iPrOH 4:1, *v*/*v*; flow rate 0.5 mL/min), mp = 125 °C; R*_f_* = 0.3 (acetone/Et_2_O 1:1, *v*/*v*).
^1^H NMR (200 MHz, CDCl_3_): *δ* = 8.68 (bs, 1H), 8.28 (d, 1H, ^3^*J*_H-H_ = 8.0 Hz), 7.53–7.74 (m, 3H), 2.94 (s, 3H).
^13^C NMR (50 MHz, CDCl_3_): *δ* = 144.7, 136.6, 131.1, 131.0, 130.7, 128.4, 123.8 (q, ^1^*J*_C-F_ = 288.3 Hz), 79.1 (h, ^2^*J*_F-C_ = 30.0 Hz), 44.7.
^19^F NMR (188 MHz, CDCl_3_): *δ* = −74.11–−73.85 (m), −74.53–−74.29 (m), −74.81 (q, ^4^*J*_F-F_ = 9.8 Hz),
IR (KBr): 3487 (O-H stretch), 1421 (O-H bend), 1130 (C-F stretch), 1066 (S=O sulfoxide), 704 (C-S stretch) cm^−1^.
MS (CI): *m*/*z* (%) = 307.0 (100) [M + H]^+^.
HRMS (EI): *m*/*z* calcd for C_10_H_8_SO_2_F_6_ 306.014920, found 306.014650 (Δ 0.88277).
(-)-(*S*)-1,1,1,3,3,3-hexafluoro-2-[*o*-(*t*-butyl-sulfinyl)phenyl]propan-2-ol (3)

The synthesis was carried out according to the general procedure with use of the following: *n*-BuLi solution in hexane (2.5 M, 3.20 mL, 8.0 mmol), TMEDA (0.24 mL, 1.6 mmol, 20%mol with respect to *n*-BuLi), perfluorocumyl alcohol **5** (680 mg, 2.8 mmol) solution in dry THF (2 mL), (-)-(*S*)-*O*-diaceton-*D*-glucosyl *t*-butanesulfinate **9** (505 mg, 1.4 mmol) solution in dry THF (5 mL), aqueous solution of H_2_SO_4_ (5%, 10 mL) and H_2_O (30 mL). After addition of sulfinate, the reaction time was prolonged to 72 h. The reaction mixture after quenching was extracted with Et_2_O (8 × 20 mL); organic layers were combined, washed with H_2_O, and dried over MgSO_4_. The solvent evaporation furnished crude product as an orange oil (903 mg). After purification using column chromatography, [SiO_2_; Et_2_O/petroleum ether 1:2, *v*/*v* (24 × 20 mL), Et_2_O/petroleum ether 1:1, *v*/*v* (12 × 20 mL), Et_2_O (5 × 20 mL)] followed by crystallization from Et_2_O solution, a white solid was obtained (34 mg; 7%).

${\left[\alpha \right]}_{D}^{298}$ = −159.9 (*c* 0.88, DCM); ee > 99% (HPLC; Chiralpak AS; hexane/iPrOH 9:1, *v*/*v*; flow rate 0.5 mL/min), mp = 132 °C; R*_f_* = 0.1 (Et_2_O/petroleum ether 1:1, *v*/*v*).
^1^H NMR (200 MHz, CDCl_3_): *δ* = 9.39 (bs, 1H), 7.93 (d, 1H, ^3^*J*_H-H_ = 7.7 Hz), 7.58–7.62 (m, 3H), 1.32 (s, 9H).
^19^F NMR (188 MHz, CDCl_3_): *δ* = −71.92 (q, 3F, ^4^*J*_F-F_ = 8.9 Hz), −75.27 (q, 3F, ^4^*J*_F-F_ = 8.9 Hz),
MS (CI): *m*/*z* (%) = 349.0 (100) [M + H]^+^.
HRMS (CI): *m/z* calcd for C_13_H_15_SO_2_F_6_ 349.06969, found 349.06870 (Δ 2.85144).
1,1,1,3,3,3-Hexafluoro-2-[*o*-(hexadecylsulfinyl)phenyl]propan-2-ol (23)

Into a stirred-in-argon-atmosphere solution of *n*-BuLi in hexane (2.70 M, 9.30 mL, 25.0 mmol), TMEDA was added dropwise (0.57 mL, 3.75 mmol, 15%mol with respect to *n*-BuLi) at rt, and the solution was stirred for further 30 min. After this time, the mixture was cooled to −10 °C, and a solution of perfluorocumyl alcohol **5** (2.08 g, 8.50 mmol) in dry THF (5 mL) was added dropwise for 5 min. Next, the mixture was allowed to warm up to rt and stirred for 26 h. After that, the solution was cooled to −10 °C, and a solution of hexadecanesulfinyl chloride **24** (2.60 g, 8.50 mmol) in dry THF (10 mL) was added dropwise for 5 min. The obtained solution was allowed to warm up to rt and stirred for 22 h. Next, the reaction mixture was quenched with H_2_SO_4_ aqueous solution (5%; 5 mL) and H_2_O (20 mL), and the obtained two-phase system was extracted with DCM (5 × 50 mL); organic layers were combined, dried over MgSO_4_, and the solvent was evaporated. The obtained crude brownish oil was purified using column chromatography (SiO; CHCl_3_/acetone 10:1, *v*/*v*), followed by preparative thin-layer chromatography (SiO_2_; CHCl_3_/acetone 8:1, *v*/*v*), to furnish pale-yellow solid (428 mg, 10%).

^1^H NMR (200 MHz, CDCl_3_): *δ* = 9.39 (bs, 1H), 8.18 (d, 1H, ^3^*J*_H-H_ = 7.8 Hz), 7.71–7.37 (m, 3H), 3.26–3.11 (m, 1H), 2.89–2.75 (m, 1H), 1.77–1.62 (m, 4H), 1.31–1.16 (m, 24H), 0.85 (t, 3H, ^3^*J*_H-H_ = 6.2 Hz).
^13^C NMR (150 MHz, CDCl_3_): *δ* = 144.5, 130.6, 129.4, 128.3, 128.2, 126.2, 122.7 (q, ^1^*J*_C-F_ = 287.4 Hz), 80.0 (h, ^2^*J*_F-C_ = 29.6 Hz), 59.0, 32.0, 29.74, 29.73, 29.64, 29.61, 29.4, 29.1, 28.6, 23.5, 23.0, 22.7, 14.2.
^19^F NMR (188 MHz, CDCl_3_): *δ* = −73.79 (q, 3F, ^4^*J*_F-F_ = 9.0 Hz), −74.35 (q, 3F, ^4^*J*_F-F_ = 9.0 Hz).
MS (CI): *m*/*z* (%) = 517.3 (27) [M+H]^+^, 499.2 (100) [M + H − H_2_O]^+^.
Reduction and racemization procedure of compound (-)-(*S*)-3

HCl gas was bubbled through a stirred solution of sulfoxide (-)-(*S*)-**3** (6.3 mg; 0.018 mmol) in DCM (2 mL) for 5 s at rt. The resulting solution was washed with K_2_CO_3_ solution (5%), dried over MgSO_4_, and the solvent was evaporated in vacuo to give a mixture of the reduction product **11** (90%) and rac-**3** (10%), as judged via ^19^F NMR analysis and the lack of optical activity.

^19^F NMR (188 MHz, CDCl_3_): *δ* = −74.83 (q, 3F, ^4^*J*_F-F_ = 8.9 Hz), −73.79, −72.80 (q, 3F, ^4^*J*_F-F_ = 8.9 Hz)
Reduction procedure of compound (-)-(*S*)-2

HCl gas was bubbled through a stirred solution of sulfoxide (-)-(*S*)-**2** (1.5 mg; 0.005 mmol) in DCM (2 mL) for 5 s at rt. The resulting solution was washed with K_2_CO_3_ solution (5%), dried over MgSO_4_, and the solvent was evaporated in vacuo to give the reduction product, sulfide **12,** as a yellowish oil (1.4 mg, 100%) based on ^19^F NMR spectrum.

^19^F NMR (188 MHz, CDCl_3_): *δ* = −77.17

## 4. Conclusions

We presented the synthesis and characterization of three novel optically active sulfoxides: (-)-(*S*)-1,1,1,3,3,3-hexafluoro-2-[*o*-(*p*-tolylsulfinyl)phenyl]propan-2-ol **1**, (-)-(*S*)-1,1,1,3,3,3-hexafluoro-2-[*o*-(methylsulfinyl)phenyl]propan-2-ol **2** and (-)-(*S*)-1,1,1,3,3,3-hexafluoro-2-[*o*-(*t*-butyl-sulfinyl)phenyl]propan-2-ol **3**. We identified their interesting properties and confirmed the existence of spontaneous reversible transformations of these compounds into the corresponding hypervalent structures, hydroxysulfuranes **10**, **15** and **16,** on the basis of the analysis of CD, ^1^H and ^19^F NMR spectra. It constitutes the first example of sulfuranes stable in solution having a hydroxyl group at the apical position. Nevertheless, all attempts to isolate these sulfuranes failed, and in the case of **1** and **2,** the starting sulfinyl structures were recovered. For *t*-butyl derivative **3,** the unprecedented transformation into the cyclic sulfinate ester **17** in solution was observed. Two speculative mechanisms of the process were proposed, and one of them was confirmed to be correct by means of GC-MS and NMR analyses. Furthermore, during attempts to racemize **2** and **3,** we found that they were stronger oxidants in comparison with analogous aryl-alkyl sulfoxides without perfluoromethyl groups, whose property can be useful in stereochemistry, particularly for asymmetric oxidation.

## Data Availability

The data underlying this study are available in the published article and its Supplementary Materials.

## Supplementary Materials

The following supporting information can be downloaded at: https://www.mdpi.com/article/10.3390/molecules31060969/s1, NMR spectra, crystallographic data of compound (*S*)-**1**.

## References

[1] Akiba K.-Y. (1999). Chemistry of Hypervalent Compounds.

[2] Drabowicz J., Halaba G. (2000). Review on Sulphur-Containing Compounds. Rev. Heteroat. Chem..

[3] Martin J.C., Balthazor T.M. (1977). Sulfuranes. 22. Stereochemical course of an associative displacement at tetracoordinate sulfur(IV) in a sulfurane of known absolute configuration. A proposed system of nomenclature for optically active pentacoordinate species. J. Am. Chem. Soc..

[4] Kawashima T., Ohno F., Okazaki R. (1994). Synthesis, Structure, and Thermolysis of a Tetracoordinate 1,2λ^4^-Oxathietane. Angew. Chem. Int. Ed..

[5] Kawashima T. (2003). Four-membered heterocyclic compounds containing high coordinate group 16 elements. Coord. Chem. Rev..

[6] Allenmark S. (2008). Recent advances in spirochalcogenurane stereochemistry—A mini review. Chirality.

[7] Balthazor T.M., Martin J.C. (1975). Sulfuranes. XVIII. Stereochemical course of an associative nucleophilic displacement at tetracoordinate sulfur(IV). Optically active trigonal bipyramidal molecule, a chlorosulfurane. J. Am. Chem. Soc..

[8] Datta A.K., Livant P.D. (1983). Synthesis and reactivity of a cyclopropyloxy sulfurane. J. Org. Chem..

[9] Zhang J., Takahashi T., Koizumi T. (1997). Optically pure alkoxychlorosulfuranes. Synthesis and transformation to chiral sulfoxides, *N*-*p*-tosylsulfilimines, and sulfonium ylides. Heterocycles.

[10] Zhang J., Saito S., Koizumi T. (1998). Stereochemical Research on the Hydrolysis of Optically Pure Spirosulfuranes:  Efficient Synthesis of Chiral Sulfoxides with Completely Opposite Stereochemistry. J. Org. Chem..

[11] Akiba K.-Y. (2011). Studies on hypervalent compounds and synthetic work using heteroaromatic cations. Heteroat. Chem..

[12] Andersen K.K. (1962). Synthesis of (+)-ethyl *p*-tolyl sulfoxide from (−)-menthyl (−)-*p*-toluenesulfinate. Tetrahedron Lett..

[13] Fernádez I., Khiar N., Llera J.M., Alcudia F. (1992). Asymmetric synthesis of alkane- and arenesulfinates of diacetone-D-glucose (DAG): An improved and general route to both enantiomerically pure sulfoxides. J. Org. Chem..

[14] Mislow K., Green M.M., Laur P., Melillo J.T., Simmons T., Ternay A.L. (1965). Absolute Configuration and Optical Rotatory Power of Sulfoxides and Sulfinate Esters. J. Am. Chem. Soc..

[15] Perozzi E.F., Michalak R.S., Figuly G.D., Stevenson W.H., Dess D.B., Ross M.R., Martin J.C. (1981). Directed dilithiation of hexafluorocumyl alcohol—Formation of a reagent for the facile introduction of a stabilizing bidentate ligand in compounds of hypervalent sulfur (10-S-4), phosphorus (10-P-5), silicon (10-Si-5), and iodine (10-I-3). J. Org. Chem..

