# Supplementary material for: The Synthesis and Characterization of Selected Optically Active Sulfoxides Bearing Perfluorocumyl Moiety, Their Spontaneous Reversible Transformations into Higher-Valent Organosulfur Species–Bicyclic Hydroxysulfuranes, and Their Irreversible Transformation into Sultine
Source: Molecules. 2026 Mar 13;31(6):969. doi: 10.3390/molecules31060969 (PMC13029366; doi:10.3390/molecules31060969)
Supplement: Supplementary file 1 [file molecules-31-00969-s001.zip › molecules-4162781-supplementary.pdf]

<u>*Supporting Information*</u>

# The Synthesis and Characterization of Selected Optically Active Sulfoxides Bearing Perfluorocumyl Moiety, Their Spontaneous Reversible Transformations into Higher-Valent Organosulfur Species–Bicyclic Hydroxysulfuranes, and Their Irreversible Transformation into Sultine

Adrian Zajac, Ewelina Wielgus, and Józef Drabowicz

## List of content

NMR spectra……………………………………………………………………… 1

ORTEP diagram and crystallographic data of compound (*S*)-**1** ………………………... 8

## $^{1}H$, $^{19}F$ and $^{13}C$ NMR spectra:

## (-)-(*S*)-1,1,1,3,3,3-Hexafluoro-2-[*o*-(*p*-tolylsulfinyl)phenyl]propan-2-ol (1)

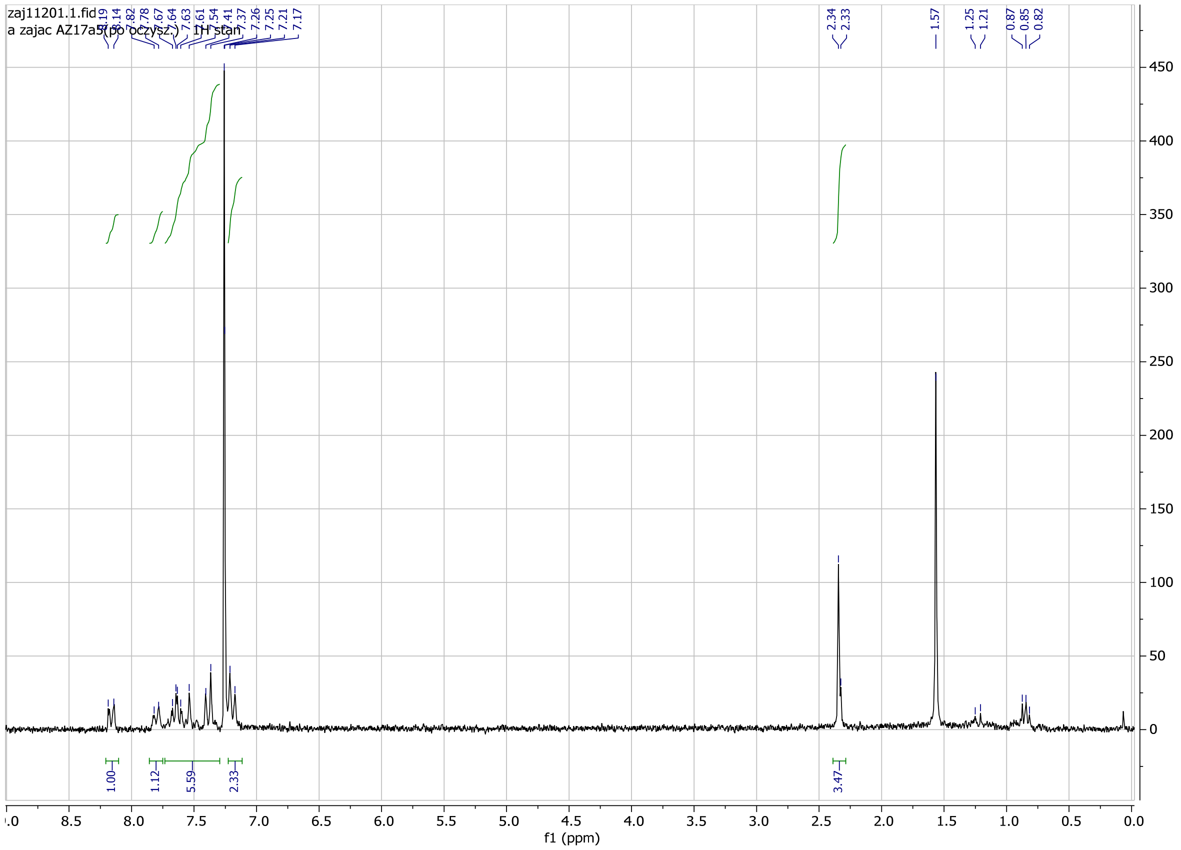

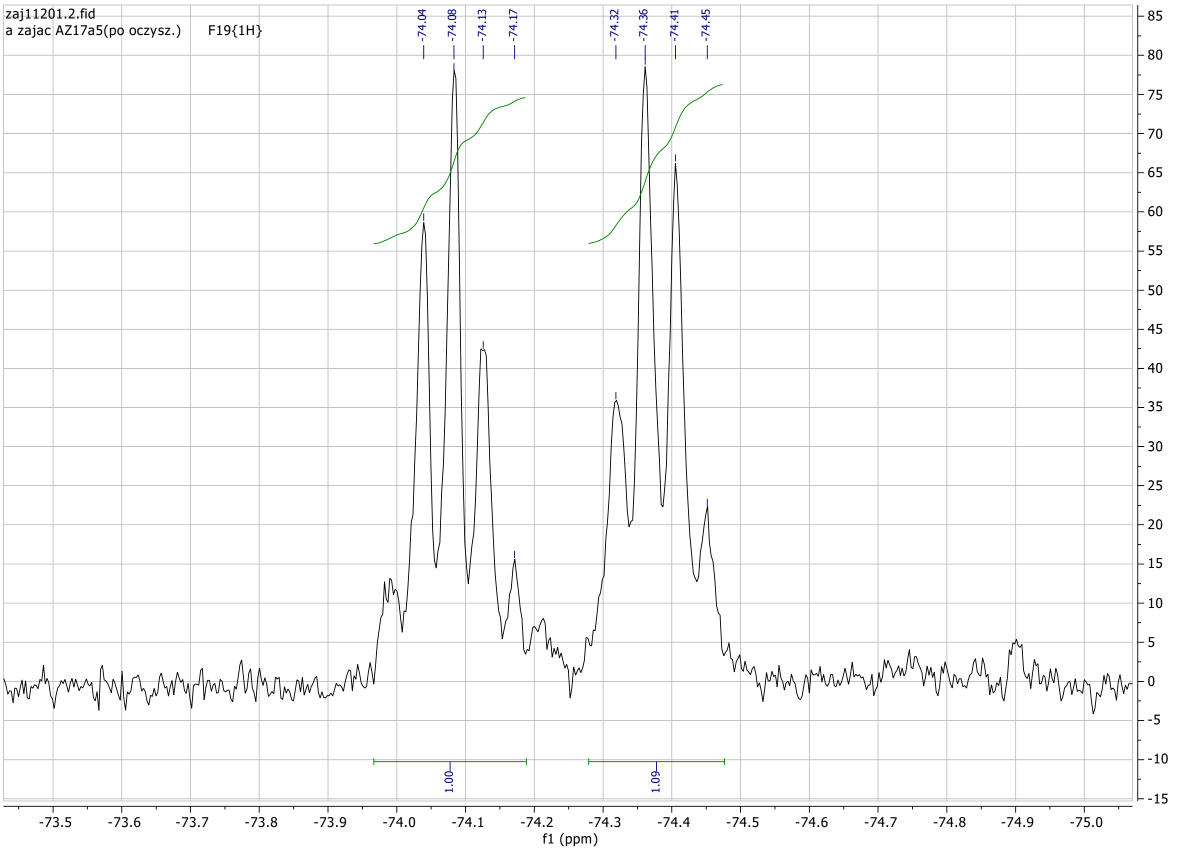
zaj11201.2.fid
a zajac AZ17a5(po oczysz.)  F19{1H}
f1 (ppm)

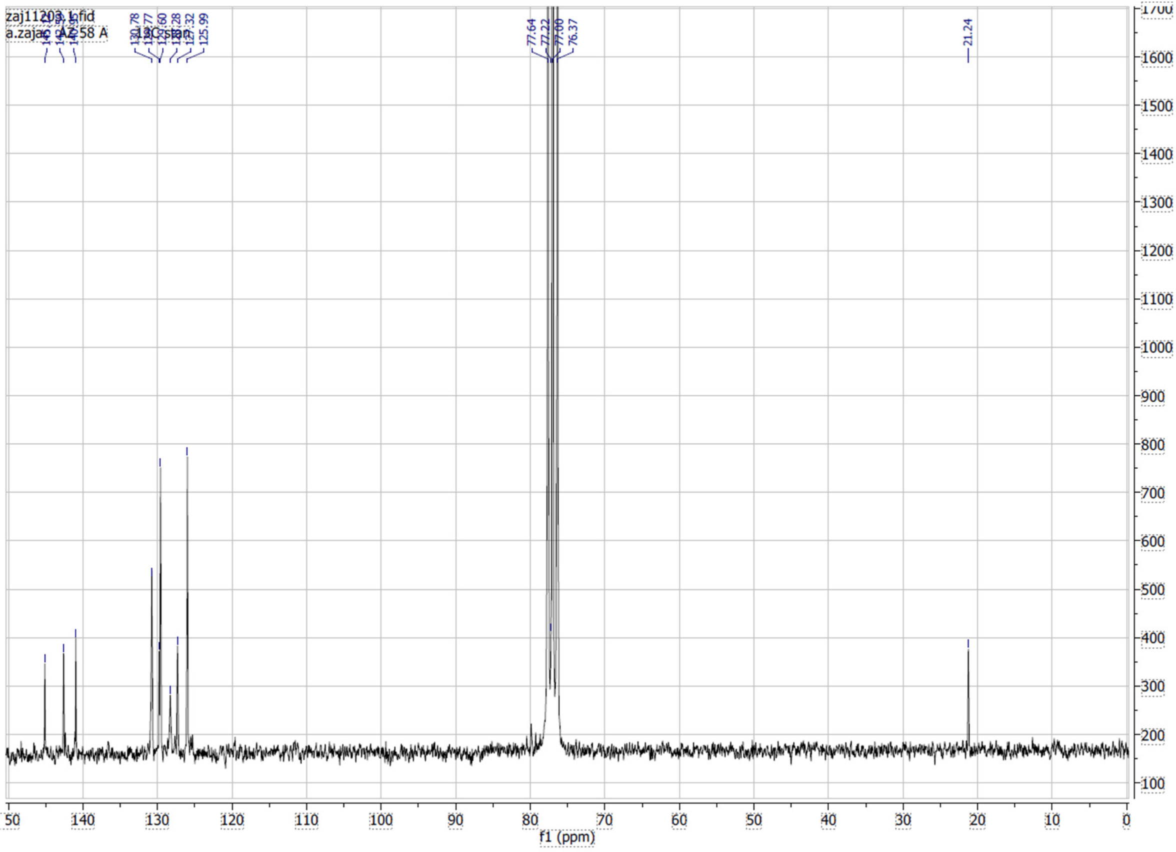
f1 (ppm)

# (-)-(*S*)-1,1,1,3,3,3-Hexafluoro-2-[*o*-(methylsulfinyl)phenyl]propan-2-ol (2)

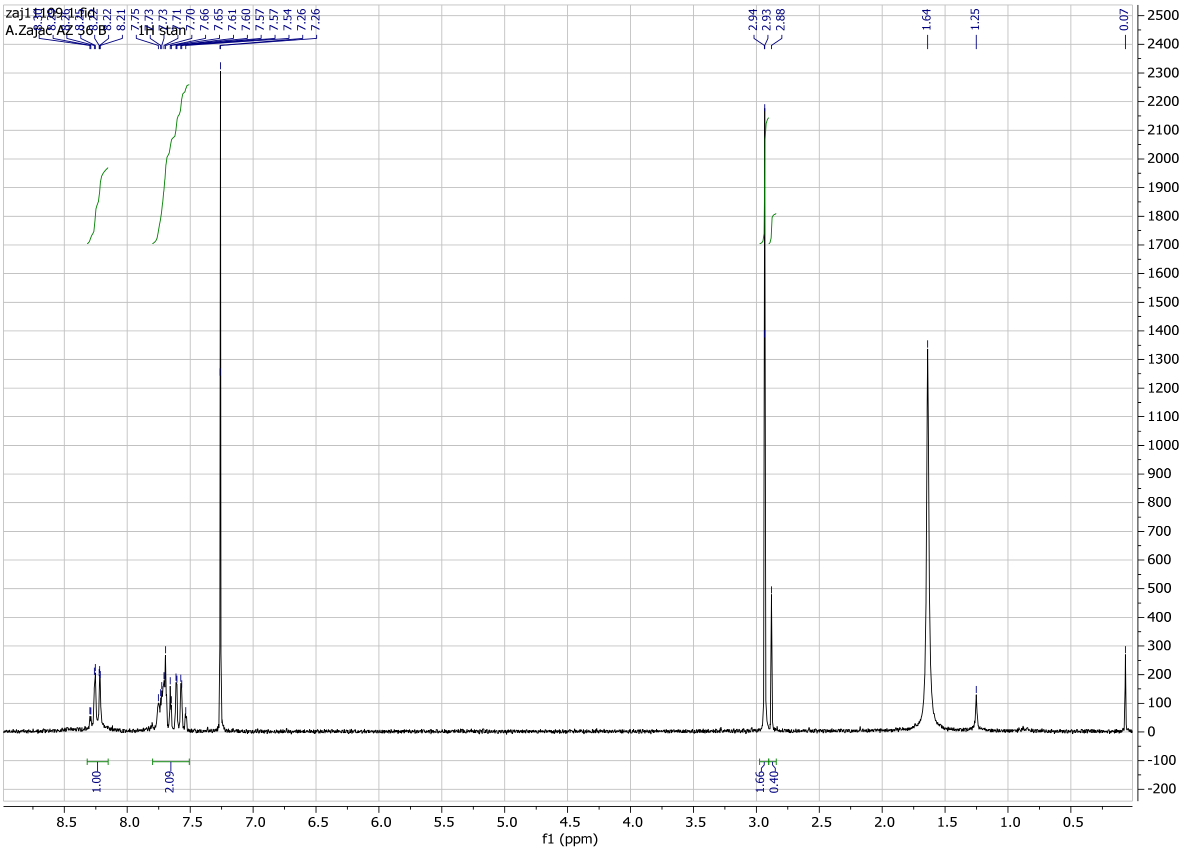


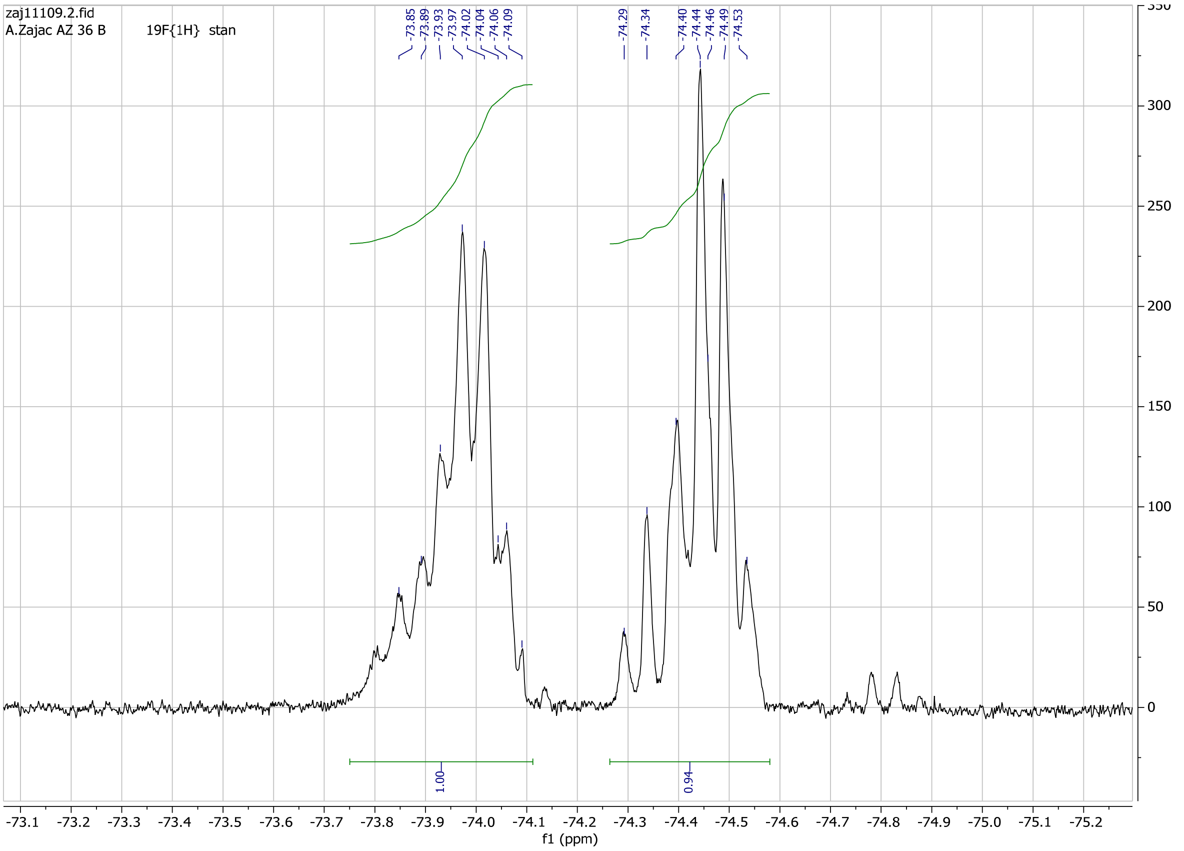

# (-)-(*S*)-1,1,1,3,3,3-hexafluoro-2-[*o*-(*t*-butyl-sulfinyl)phenyl]propan-2-ol (3)

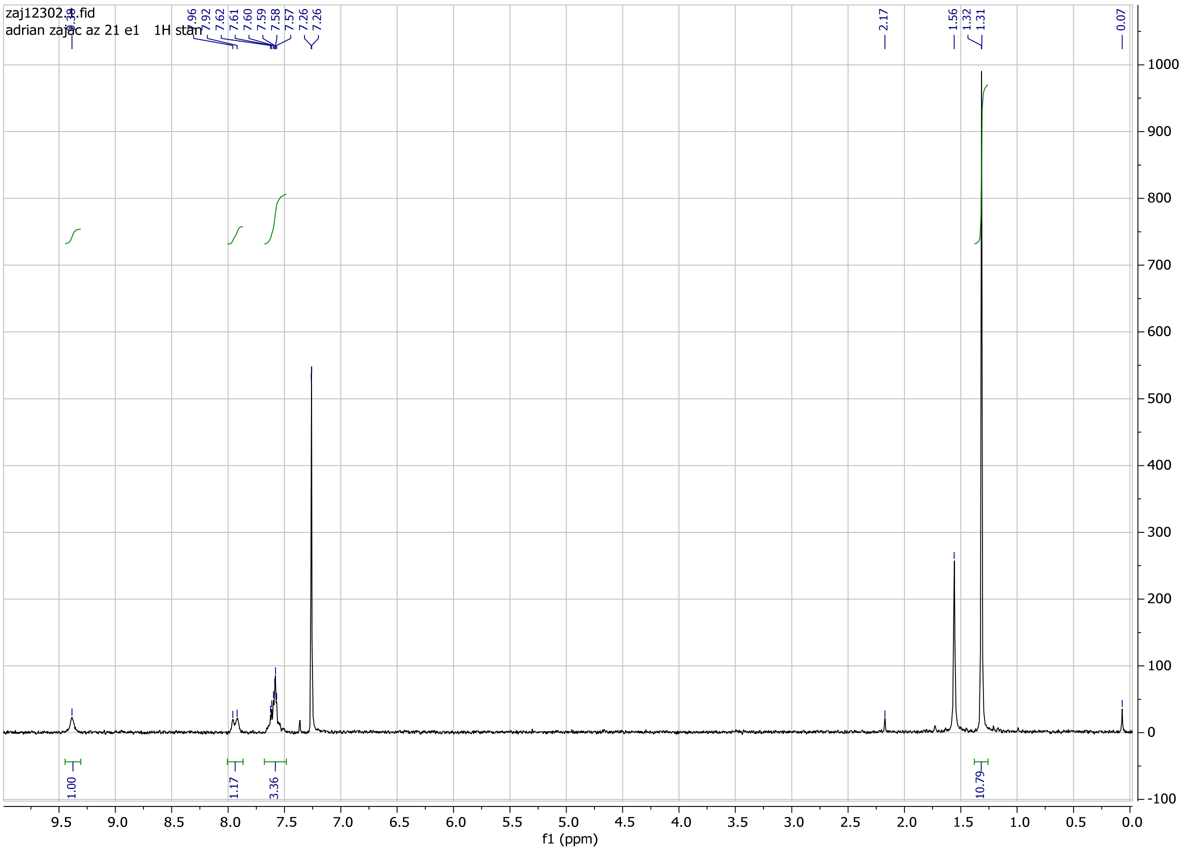

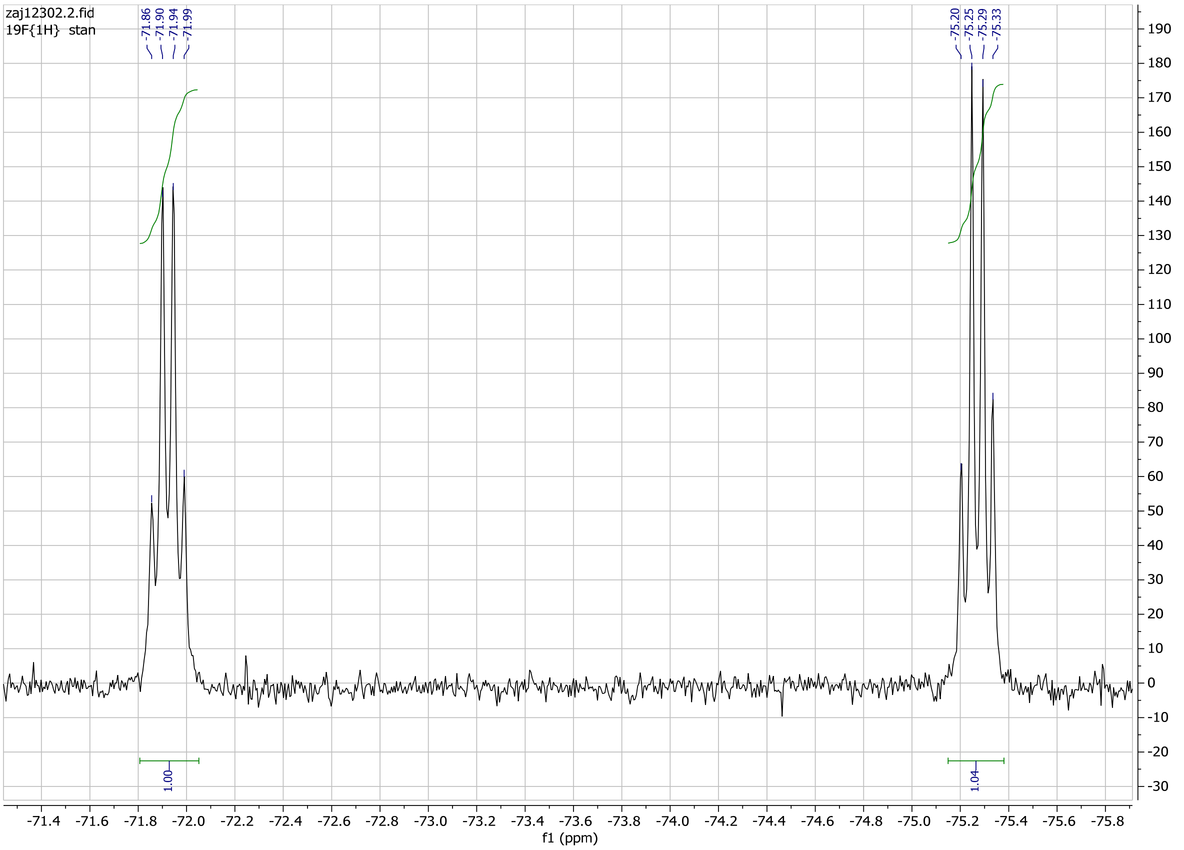

# 1,1,1,3,3,3-Hexafluoro-2-[*o*-(hexadecylsulfinyl)phenyl]propan-2-ol (23)

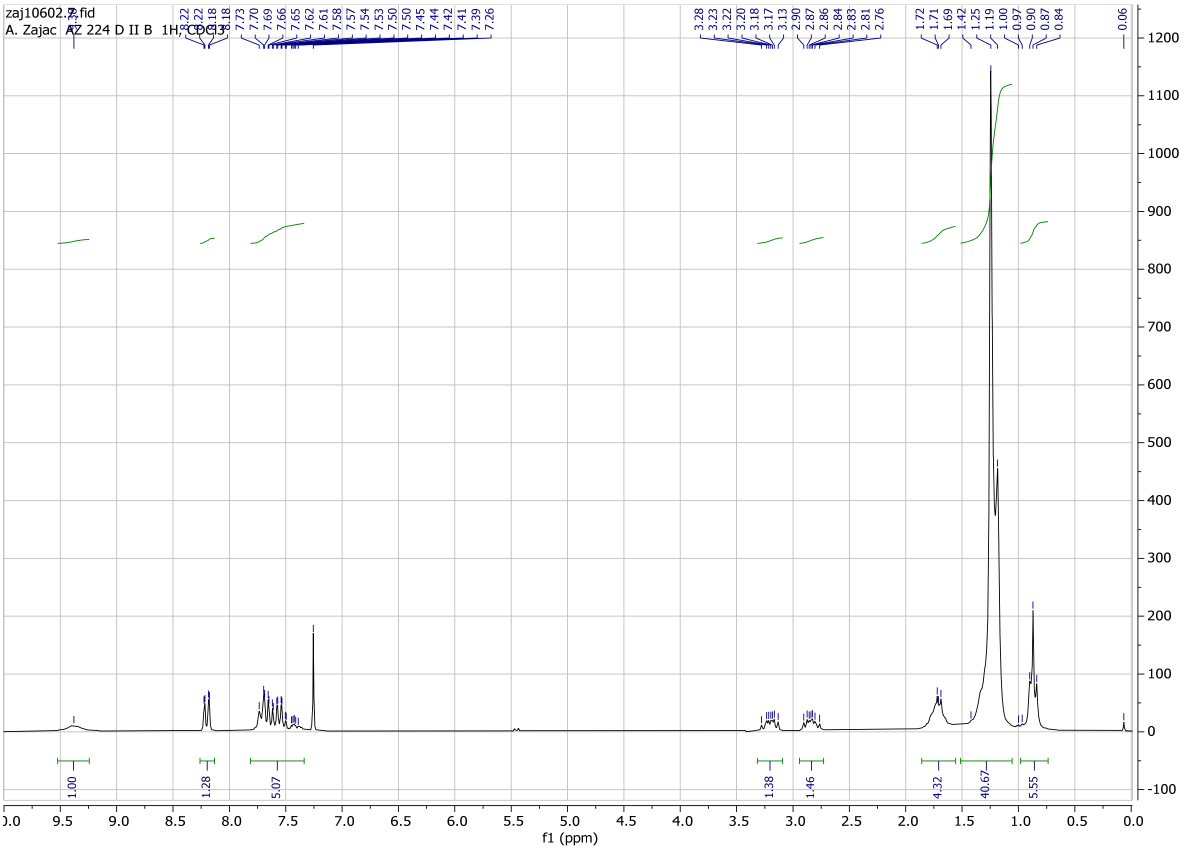


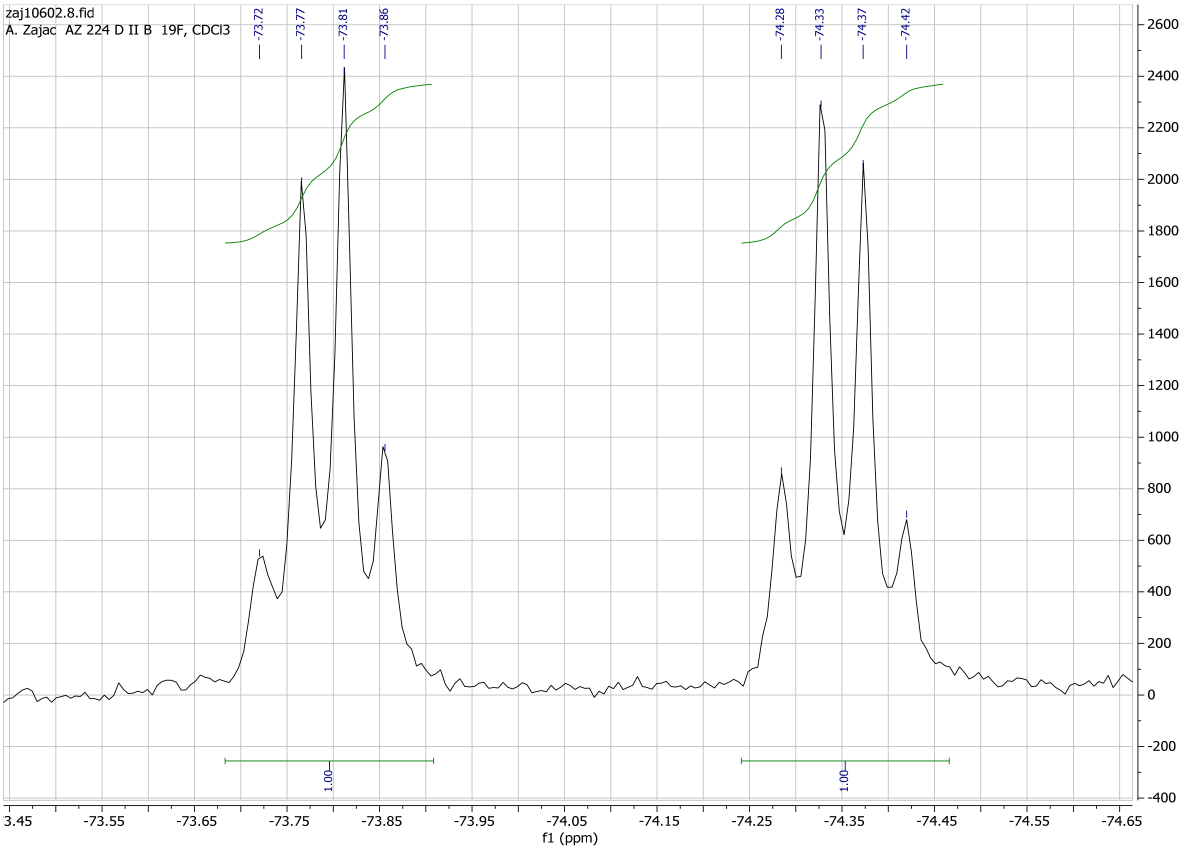

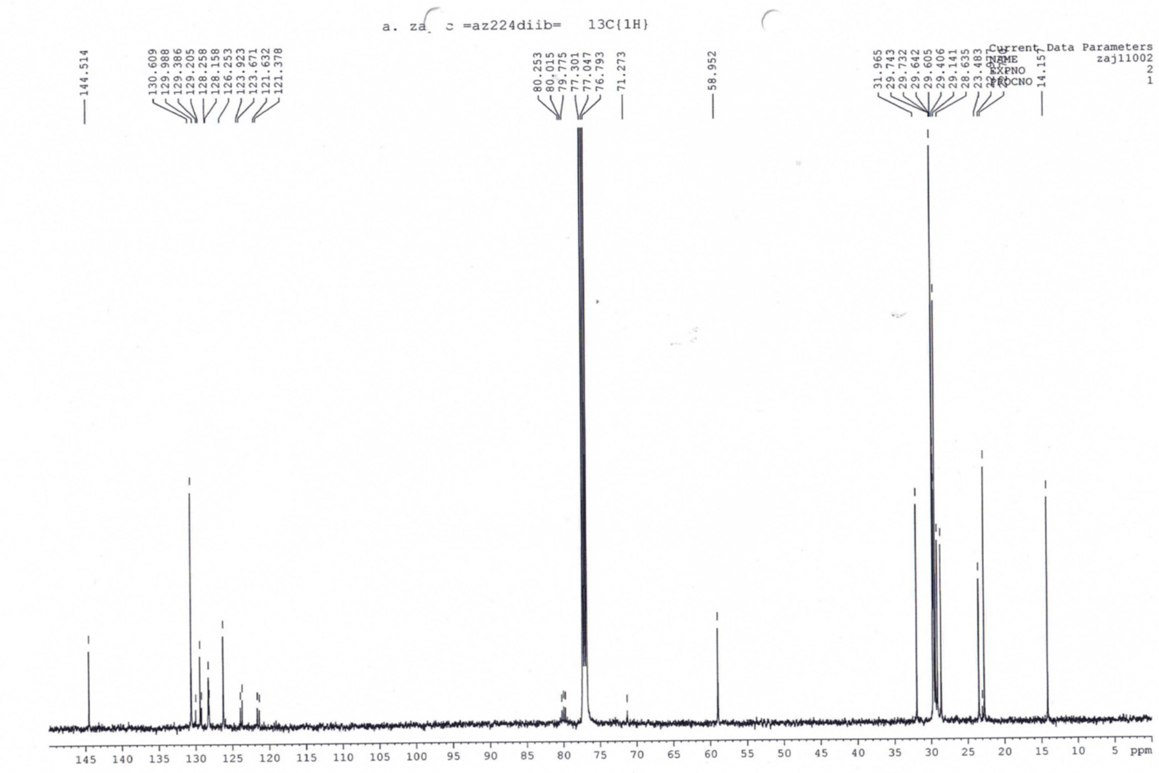

**$^{19}$F NMR spectrum of the mixture of products from reaction of (-)-(*S*)-3 with gaseous HCl**

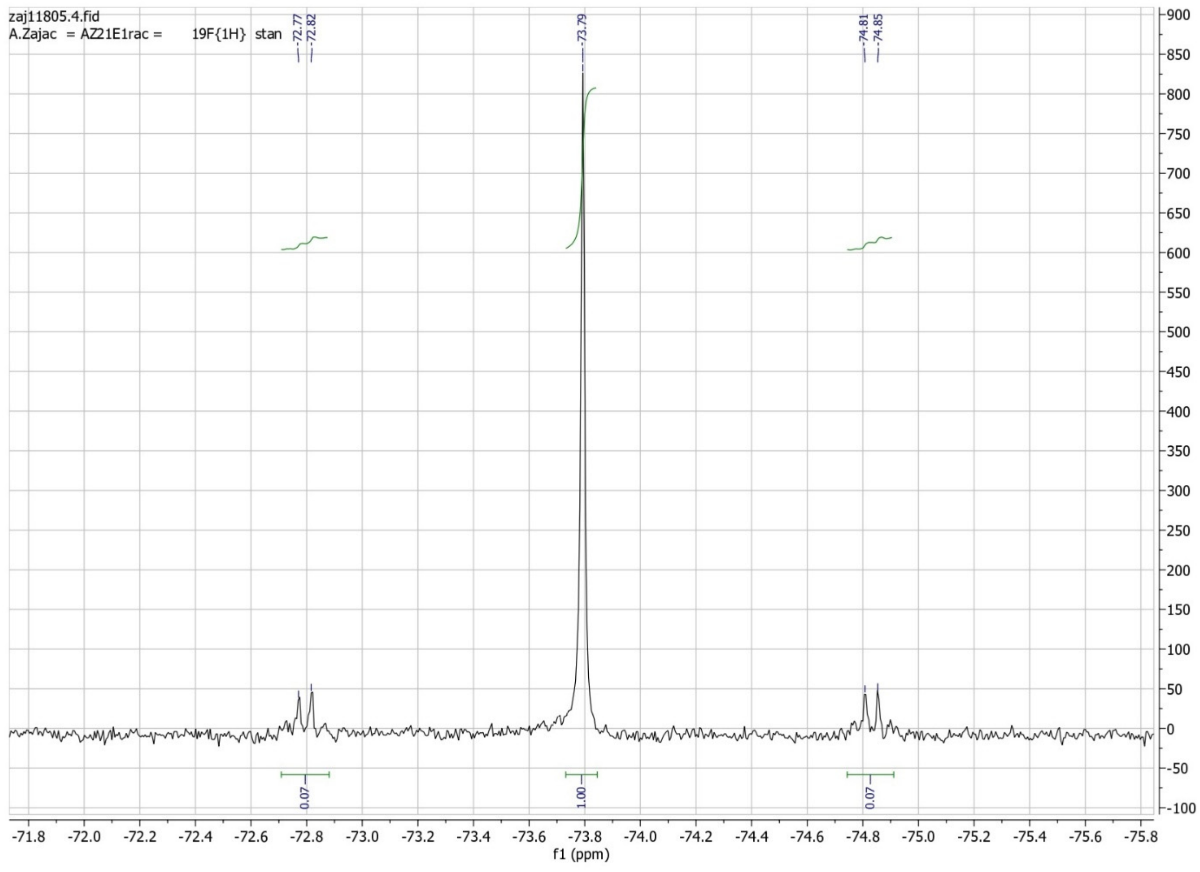

**$^{19}$F NMR spectrum of the mixture of products from reaction of (-)-(*S*)-2 with gaseous HCl**

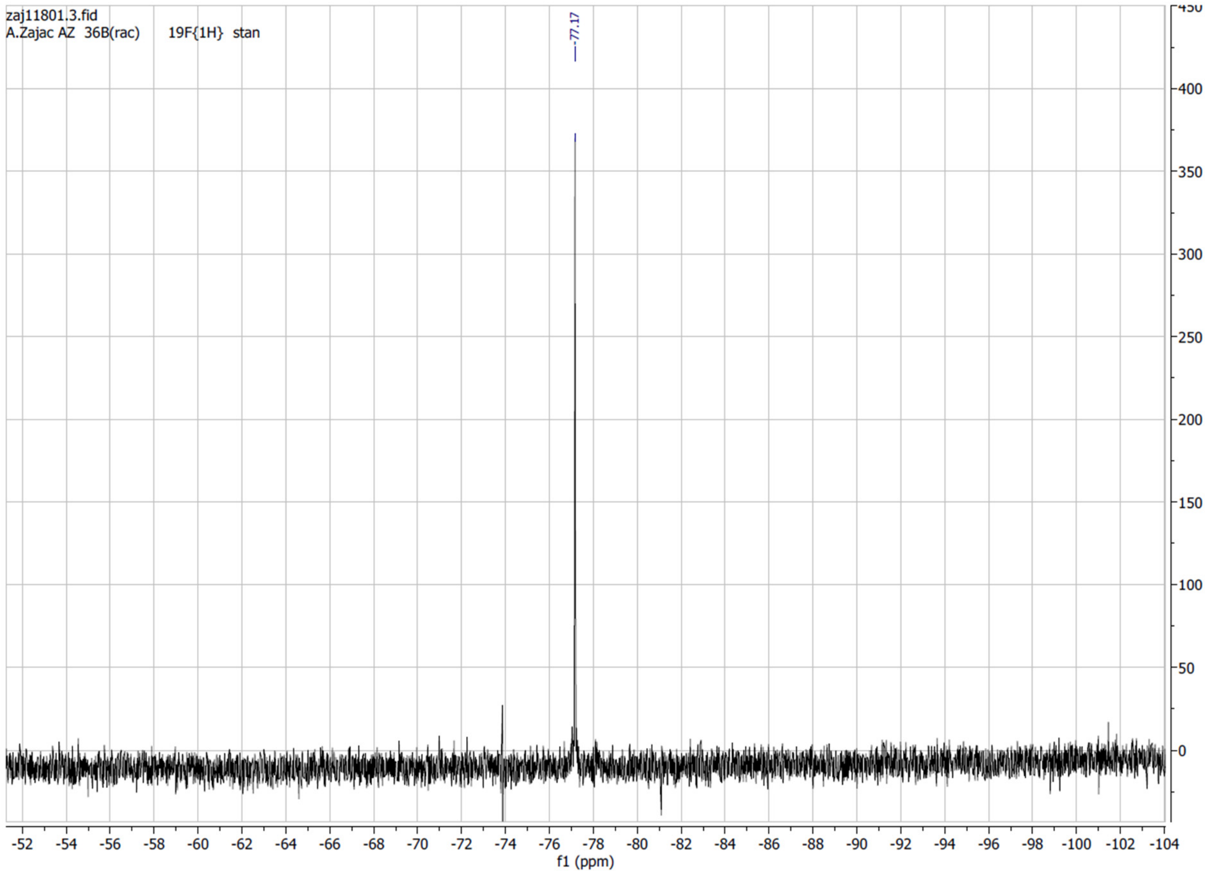

## ORTEP diagram and crystallographic data of compound (*S*)-1

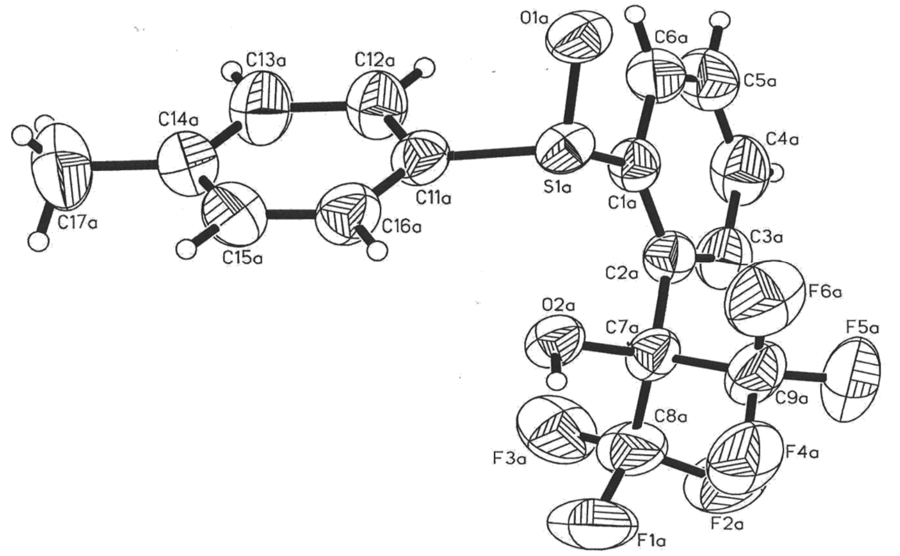


| | |
|---|---|
| Formula weight | 382.32 |
| Crystallographic system | orthorhombic |
| Space group | $P2_12_12_1$ |
| a [Å] | 13.110(3) |
| b [Å] | 13.698(3) |
| c [Å] | 29.020(6) |
| V [$Å^3$] | 5211.1(28) |
| Z | 12 |
| $R_{obs}$ | 0.0568 |
| Flack $\chi$ | 0.03(2) |
| Absolute configuration | $S_S$ |
| $T_{meas.}$ | 293(2) |
| F(000) | 2328 |
| $[\alpha]_{589}$ | - 95.8 |
